# The plasticity of pyramidal neurons in the behaving brain

**DOI:** 10.1098/rstb.2023.0231

**Published:** 2024-06-10

**Authors:** Elena Regele-Blasco, Lucy M. Palmer

**Affiliations:** ^1^ The Florey Institute of Neuroscience and Mental Health, The Florey Department of Neuroscience and Mental Health, University of Melbourne, Victoria 3052, Australia

**Keywords:** plasticity, pyramidal neurons, *in vivo*, spines, dendrites

## Abstract

Neurons are plastic. That is, they change their activity according to different behavioural conditions. This endows pyramidal neurons with an incredible computational power for the integration and processing of synaptic inputs. Plasticity can be investigated at different levels of investigation within a single neuron, from spines to dendrites, to synaptic input. Although most of our knowledge stems from the *in vitro* brain slice preparation, plasticity plays a vital role during behaviour by providing a flexible substrate for the execution of appropriate actions in our ever-changing environment. Owing to advances in recording techniques, the plasticity of neurons and the neural networks in which they are embedded is now beginning to be realized in the *in vivo* intact brain. This review focuses on the structural and functional synaptic plasticity of pyramidal neurons*,* with a specific focus on the latest developments from *in vivo* studies.

This article is part of a discussion meeting issue ‘Long-term potentiation: 50 years on'.

## 1. Introduction

One of the most important functions of the brain is to rapidly adapt to our ever-changing environment. As the computational building blocks of the brain, neurons must, therefore, be dynamic and alter their activity according to changing input patterns. This is only possible owing to the plasticity of individual neurons and the neural networks in which they are embedded. That is, neurons are malleable and capable of rapidly updating their signalling responses to changes in incoming input patterns. Revealing the plasticity of neurons, and how this influences cellular signalling and network encoding within the brain, is crucial to understanding how the brain drives one of the most important functions of all, learnt behaviour.

As the most abundant principal cell type within the cortex, hippocampus and amygdala [1–3], most learnt behaviours involving active cognition are driven, at least in part, by glutamatergic pyramidal neurons. Although there are various different subtypes of pyramidal neurons with different input sources and output targets, they are all characterized by their pyramid-shaped soma and extensive polarized dendritic arborization. The unique morphology of these neurons is beneficial for integrating synaptic input received from different pathways into a unified output. Since input patterns received during learning and the execution of learnt behaviour are typically dynamic, these integrative processes must be highly plastic. Synaptic plasticity—that is, the activity-dependent modification in the synaptic strength of neural connections—is believed to be at the core of most brain functions [4]. The most well-recognized theory of synaptic plasticity is the Hebbian theory. Coined by Donald Hebb, Hebbian plasticity is defined as ‘the persistence or repetition of a reverberatory activity that tends to induce lasting cellular changes’ [5]. Long-term potentiation (LTP) and long-term depression (LTD) are two forms of Hebbian plasticity that can last from minutes to hours [6]. An extension of these classic and much-studied plasticity modes is spike-timing-dependent plasticity (STDP). Involving backpropagating action potentials, STDP acts as a coincidence detector between synaptic input and somatic (action potential) output [7,8]. STDP is, therefore, dependent on dendritic location [9–12], where the relative timing of neural input and output is influenced by the dendritic location of the synaptic input. Since learning typically requires the temporal association of specific inputs with an active action potential output, STDP is commonly believed to be a crucial plasticity mechanism that drives learnt behaviour.

**Figure 1.**
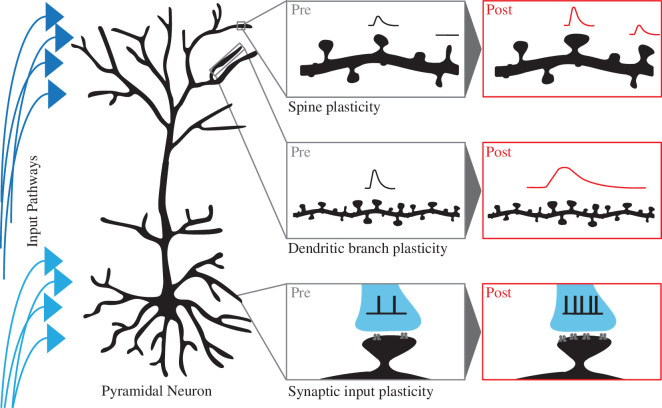
The different levels of neural plasticity. Neural signalling can be assessed before (pre, grey) and after (post, red) plasticity at the level of spines, dendrites and axonal input. (i) Spine plasticity: spine morphology is plastic, causing a change in the synaptic response in spines (red). (ii) Dendritic branch plasticity: dendritic integration is plastic and can result in changes in the voltage response (red). (iii) Synaptic input plasticity: axonal action potential input patterns (analogue input) are plastic, which can result in changes in post-synaptic receptor densities (grey).

Despite being of vital importance, we only have a limited understanding of how, and when, plasticity occurs *in vivo*. This is largely owing to both the sheer complexity of the brain functions involved as well as limitations in our capability of measuring small changes in neural encoding. In this review, we delve into the plasticity of neural signalling within a single pyramidal neuron, with a focus on the latest developments from *in vivo* studies (figure 1). First, we review plasticity in dendritic spines from changes in morphology and density to how spine signalling changes with changing input and output patterns in the intact brain. We then discuss the plasticity of signalling within dendritic branches, and how dendritic plasticity can be compartmentalized to increase the computational capabilities of a single neuron. We next review the plasticity of input pathways, and finally, we discuss plasticity occurring at behavioural timescales. This review will focus on the plasticity of single pyramidal neurons in the awake–behaving brain and will not necessarily provide a thorough review of all modes of plasticity. Owing to their dominance in research efforts, we will primarily focus on the plasticity of pyramidal neurons within the hippocampus and cortex, and although different subtypes of pyramidal neurons reside in these areas, plasticity within pyramidal neurons as a collective class of neurons is considered. It should be noted that pyramidal neurons, both between and within brain regions, are not all the same and they may have different plasticity mechanisms that are not detailed in this review.

## 2. Dendritic spine plasticity *in vivo*

When one thinks of plasticity, we often think about changes that occur at the site of synaptic input, that is, dendritic spines. Since they were first described by Santiago Ramón y Cajal in the nineteenth century, the functional role of dendritic spines and how they shape the activity of neurons has been a source of intrigue. Owing to their sub-micron size, most of what we know about spine plasticity stems from experiments conducted within the *in vitro* brain slice preparation. However, with the advances in experimental techniques that can probe neural function at the sub-micron level in the intact brain, we are now able to measure the activity of dendritic spines in response to changes in the sensory environment in the intact brain. Known to undergo activity-independent [13] and activity-dependent [14,15] plasticity, dendritic spines have been shown to dramatically increase in size following sensory deprivation [16,17] and the entire spine apparatus has been shown to dynamically appear or disappear in an experience-dependent manner [14,18–23]. Thought to be owing to changes in patterns of synaptic input [24,25], these changes in spine morphology—and specifically, the size of the spine neck—have a dramatic influence on synaptic voltage responses [26–30]. The plasticity of spine morphology is often correlated with changes in the receptors within the spine head [31]. For example, both NMDA [32,33] and AMPA [34,35] receptors have been shown to have increased trafficking and spine density following plasticity induction. These changes in spine dynamics lead to changes in synaptic strength [17,31] and are thought to play a vital role in learning and memory [32,36].

Despite being shown to be highly plastic and adaptable to changing input patterns, the dynamics of spine signalling during learnt behaviour are only just beginning to be realized. Rapid changes in spine morphology are thought to provide a structural basis for experience-driven changes in neural activity [15,23]; however, further assessment of the structural and functional plasticity of spines *in vivo* is needed to truly grasp the importance of spine plasticity in shaping neural responses within the naturally dynamic environment.

## 3. Dendritic branch plasticity *in vivo*

**Figure 2.**
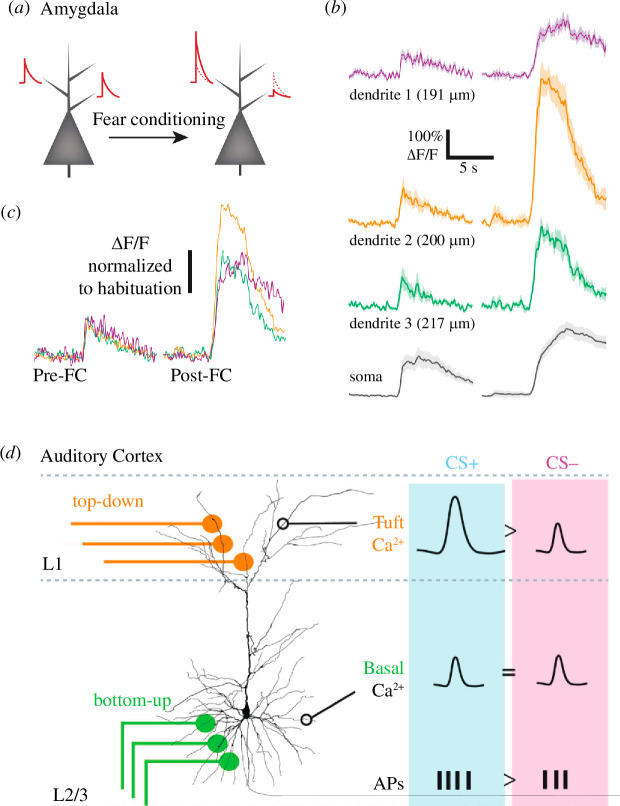
Examples of dendritic plasticity following fear learning. During fear conditioning, a tone-conditioned stimulus is paired with an aversive unconditioned stimulus (CS^+^) and an unconditioned stimulus is presented alone (CS^−^). (*a*) Simultaneous two-photon calcium imaging from soma and dendrites of pyramidal neurons within the lateral amygdala before (left) and after (right) auditory fear conditioning. (*b*) Calcium responses (ΔF/F) to CS^+^ stimulus recorded from three dendritic segments and soma from a neuron before and after fear conditioning. (*c*) Overlay of dendritic responses before (left) and after (right) fear conditioning (FC). (*d*) Two-photon calcium imaging was performed from tuft and basal dendrites of L2/3 pyramidal neurons within the auditory cortex following fear conditioning. Tuft dendritic responses to CS^+^ were larger than CS, whereas fear conditioning had no influence on calcium responses in basal dendrites. CS^+^ lead to more action potentials (APs). (*a–c*) Modified from [41]. (*d*) Modified from [12].

Often described as the fundamental functional unit in the brain [37], dendrites provide neurons with an ideal substrate for both activity- and input-dependent plasticity [38]. It is therefore not surprising that dendritic plasticity has been reported in pyramidal neurons within various brain regions *in vivo,* including the motor cortex [39], auditory cortex [12], hippocampus [40] and lateral amygdala [41] (figure 2). Typically occurring hand-in-hand with spine plasticity, plasticity can result in changes to both dendritic branching morphology [42] and/or dendritic signalling [43]. For example, the induction of LTP in hippocampal CA1 pyramidal neurons is accompanied by a local increase in dendritic excitability [43]. Various mechanisms have been shown to underlie local dendritic plasticity, including (i) local protein synthesis [44–46], (ii) ion channel density and distribution [47–49], (iii) contribution of intracellular calcium stores [40,50,51], (iv) intrinsic excitability [52–54] and (v) patterns of synaptic input [3,22,55,56]. In addition to influencing local dendritic signalling, dendritic plasticity can also strongly impact overall neural excitability. Take, for example, the generation of dendritic spikes, which are large supralinear dendritic voltage events [57] that directly influence somatic firing [58]. Plasticity that results in lowering the voltage threshold for the generation of dendritic spikes can lead to a direct increase in somatic action potentials [59,60], which is an exciting topic of ongoing research [61].

The spatial targeting of different input pathways within both the cortex [62–64] and hippocampus [65] makes dendrites an ideal candidate for gating specific input pathways. This was observed following fear learning, where dendritic plasticity occurred in one dendritic region but not another [12]. This compartmentalization of dendritic plasticity may be important in enabling the simultaneous [66,67] or individual [67] processing of different synaptic input streams within a single pyramidal neuron [68]. The independent plasticity of multiple computational units within a single neuron may ultimately act to enhance the computational power of pyramidal neurons [69]. Interestingly, human pyramidal neurons have been shown to have greater dendritic compartmentalization than the more commonly studied rodent cortical neurons [70]. In combination with their larger dendritic arbours [71], distinct biophysical composition [72] and highly plastic neurons [73–75], this may provide human neurons with enhanced computational abilities and specific dendritic integrative properties [76–78]. Taken together, signalling within individual dendritic branches is plastic *in vivo*, which may serve to enhance specific input streams and increase the computational capabilities of a single pyramidal neuron.

## 4. Synaptic input plasticity *in vivo*

Since most learnt behaviours involving active cognition are driven, at least in part, by pyramidal neurons, these glutamatergic neurons must effectively integrate synaptic input into action potential output. The integrative properties of pyramidal neurons are principally determined by the structure of the brain region in which they reside. Take for example cortical pyramidal neurons. The cortex is a highly hierarchical and conserved laminar organization consisting of six layers [79]. Within sensory cortices, external sensory thalamic (feed-forward; bottom-up) information targets layer 4, whereas long-range internally generated feed-back (top-down) information typically targets the superficial layer 1 [80]. Similarly, CA1 pyramidal neurons within the hippocampus also receive spatially defined synaptic input, receiving input from the Schaffer collateral pathway stemming from the CA3 hippocampal region and perforant pathway from the entorhinal cortex [3,65]. Likewise, pyramidal neurons in the amygdala receive spatially defined feed-forward and feed-back input from the thalamus, striatum and cortex [81,82]. Distinct top-down and bottom-up brain connectivity has also been illustrated in humans [83], suggesting it is a powerful connectivity paradigm for optimized brain function within the mammalian brain, including humans. Dynamic changes in these different input pathways can rapidly modify neural responses to incoming information, enabling neurons to quickly alter the processing of sensory inputs [12,84] and learnt behaviour [3]. Take, for example, synaptic input from higher-order thalamic nuclei to cortical neurons. This pathway has been shown to be highly plastic and undergo experience-dependent modifications during sensory learning [85–87] and planning of sensory-cued motor responses [88]. Input from the perirhinal cortex to the sensory cortex has also been shown to be important during learning, resulting in learning-related plasticity of sensory encoding within pyramidal neurons in the primary somatosensory cortex [89].

The plasticity of synaptic input has been shown to play a crucial role in gating the learning of many different behaviours, including the highly studied sensory association. Rapidly updating the pattern of synaptic input according to learnt behaviour provides an effective plasticity mechanism to alter the activity of either single neurons or populations of neurons within the brain.

## 5. Behaviourally relevant synaptic plasticity *in vivo*

**Figure 3.**
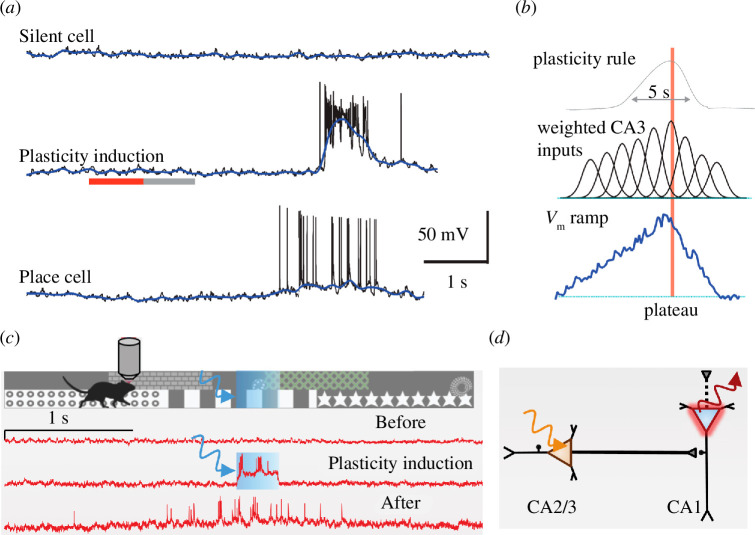
Behavioural timescale synaptic plasticity (BTSP**.** (*a*) Voltage in a silent CA1 pyramidal neuron (i) preceding, (ii) during and (iii) after the generation of a calcium plateau potential forming a place cell. (*b*) CA1 neurons receive unpotentiated, tuned CA3 excitatory inputs. (i) A long-lasting plasticity rule controls the (ii) synaptic weights of CA3 inputs. (iii) Plasticity alters the weight of the tuned inputs that generate a voltage ramp in CA1 pyramidal neurons. (*c*) All-optical modulation/readout of membrane voltage in hippocampal BTSP in virtual reality. Photoactivation of CA2/3 inputs induces plasticity in CA1 neurons in mice navigating a virtual environment. This leads to the induction of place fields in single CA1 cells. (*d*) TActivity in presynaptic CA2/3 cells is required for the induction of plasticity in CA1 neurons. (*a,b*) Modified from [59]. (*c,d*) Modified from [93].

At its centre, Hebbian plasticity, notably STDP, relies heavily on the causality and activity repetition of synaptically coupled neurons [7,11,90]. It requires the learnt association of specific inputs to be paired with an active output, and it is this correlation that has widely been considered to underlie learning and memory. However, if we consider that these standard plasticity rules operate under the timescale of milliseconds, we quickly realize that these rules can be limiting during natural behaviour, which typically occurs at the timescale of seconds to minutes. Ultimately, to explain the dynamic encoding that occurs during behaviour, plasticity rules that include more than a tight temporal correlation of casual input and output activity are needed. A new form of synaptic plasticity was recently described that does not follow the Hebbian rule of learning. Termed ‘behavioural timescale synaptic plasticity’, or BTSP, this plasticity mode operates on longer timescales, rather than the standard rule of milliseconds, which are more in line with how we typically interact with our environment [59]. BTSP is unique as it does not require correlated input and output and can induce plasticity within a single trial—characteristics that differ from the Hebbian plasticity requirements. First described in hippocampal CA1 neurons, it was shown that place fields could be formed by BTSP [59]. Here, following the initiation of a large subthreshold voltage event (calcium plateau potential) within the distal apical dendrites of silent CA1 neurons, a long-lasting depolarization spreads throughout the neuron, inducing potentiation of synaptic inputs [59]. This, in turn, generates a slow voltage ramp, which subsequently evokes location-specific place field firing [91,92] (figure 3*a,b*). Since its discovery, BTSP has also been shown to occur during different behavioural conditions. Within a virtual-reality environment, optogenetic activation of CA1 neurons generated a stable neural representation of activated locations and synaptic input from CA2/3 was potentiated [93]. Furthermore, it was shown that activity in presynaptic CA2/3 cells is required for the induction of place fields in CA1 neurons (figure 3*c,d*). BTSP can also reshape existing fields via bidirectional synaptic weight changes according to the temporal proximity of calcium plateau potentials to pre-existing place fields [94]. Interestingly, it appears as though BTSP might depend inversely on synaptic input because plateau potentials evoked near an existing place field create less potentiation.

Overall, BTSP provides a cellular mechanism whereby the neural encoding is shaped by behavioural experience, demonstrating that prior behaviour modifies subsequent behaviour. We are just at the tip of the iceberg in our understanding of behaviourally relevant plasticity, and there are now more questions than we have answered. BTSP creates new correlations and changes existing ones, altering synaptic responses during behaviour, including signalling that occurs before and after a behavioural event [95]. It is highly possible that BTSP is the basis of most behaviours, although the exact parameters may be specific to the needs of the behaviour itself.

## 6. Concluding remarks

Synaptic plasticity is crucial to learning and behaviour. It comes in all shapes and sizes, and it appears as though all signalling mechanisms, from the site of synaptic input to the input itself, are plastic. Due to experimental constraints, different levels of investigation are typically looked at in isolation. However, in reality, they are all interlinked and the plasticity of one signalling mechanism would lead to changes, and plasticity, in another. For example, spine plasticity can be caused by synaptic input plasticity, which leads to dendritic plasticity. Plasticity mechanisms are often hard to tackle in the experimental setting, where a single neuron can receive thousands of different synaptic inputs that result in a cascade of signalling mechanisms within the dendritic spine. Important work has been undertaken using computational models where general plasticity models of synaptic plasticity within pyramidal neurons have been established [96–99]. These models illustrate that just a few parameters are sufficient to induce synaptic plasticity, which brings to question whether this is also true of neurons in the intact brain. How does plasticity manifest and contribute to learnt behaviour? How long does it last? How do the different plasticity rules interplay in single neurons during behaviour? Answering these questions, and more, requires an interplay between experimental, theoretical and computational approaches.

We are just beginning to understand the plasticity mechanisms within the behaving brain. This review specifically focuses on the plasticity of pyramidal neurons *in vivo*. This is, of course, a very limited scope as there are many other cell types that contribute to the flexibility of the brain. Take for example interneurons. Inhibition within neural networks serves to balance and restrict the spread of excitation [100,101], organize temporal and spatial patterns of neural activity [102–104] and shape the response of neurons [105–107]. Indeed, interneurons have been shown to change their signalling [108,109] and have a strong influence on learning [110,111]. Altering the impact of inhibition via interneuron plasticity can have a drastic, and immediate, influence on neural networks. Another consideration of *in vivo* plasticity involves ‘metaplasticity’. That is, the ‘plasticity of synaptic plasticity’ [112]. Previous behavioural experiences result in continual changes in neural encoding that can have an effect on the induction of future plasticity events [112,113]. These persistent changes could, for example, prime a neuron for learning [54,114] and recent evidence suggests that metaplasticity is involved in processes that underlie learning and memory in the cortex *in vivo* [115].

Plasticity is a dynamic process and it contributes to the overall flexibility of neural encoding in the behaving brain. Through more focused research, we will have a greater understanding of the plastic brain, how it adapts to the demands of our surrounding environment and shapes the immense computational capability of individual neurons.

## Data Availability

This article has no additional data.
